# Vitamin K as a Powerful Micronutrient in Aging and Age-Related Diseases: Pros and Cons from Clinical Studies

**DOI:** 10.3390/ijms20174150

**Published:** 2019-08-25

**Authors:** Dina C. Simes, Carla S. B. Viegas, Nuna Araújo, Catarina Marreiros

**Affiliations:** 1 Centre of Marine Sciences (CCMAR), Universidade do Algarve, 8005-139 Faro, Portugal; 2 GenoGla Diagnostics, Centre of Marine Sciences (CCMAR), Universidade do Algarve, 8005-139 Faro, Portugal

**Keywords:** vitamin K, vitamin K-dependent proteins, cardiovascular diseases, skeletal health, inflammaging, pathological calcification, inflammation

## Abstract

Vitamin K is a multifunctional micronutrient implicated in age-related diseases such as cardiovascular diseases, osteoarthritis and osteoporosis. Although vitamin K-dependent proteins (VKDPs) are described to have a crucial role in the pathogenesis of these diseases, novel roles have emerged for vitamin K, independently of its role in VKDPs carboxylation. Vitamin K has been shown to act as an anti-inflammatory by suppressing nuclear factor κB (NF-κB) signal transduction and to exert a protective effect against oxidative stress by blocking the generation of reactive oxygen species. Available clinical evidences indicate that a high vitamin K status can exert a protective role in the inflammatory and mineralization processes associated with the onset and progression of age-related diseases. Also, vitamin K involvement as a protective super-micronutrient in aging and ‘inflammaging’ is arising, highlighting its future use in clinical practice. In this review we summarize current knowledge regarding clinical data on vitamin K in skeletal and cardiovascular health, and discuss the potential of vitamin K supplementation as a health benefit. We describe the clinical evidence and explore molecular aspects of vitamin K protective role in aging and age-related diseases, and its involvement as a modulator in the interplay between pathological calcification and inflammation processes.

## 1. Introduction

**Figure 1:**
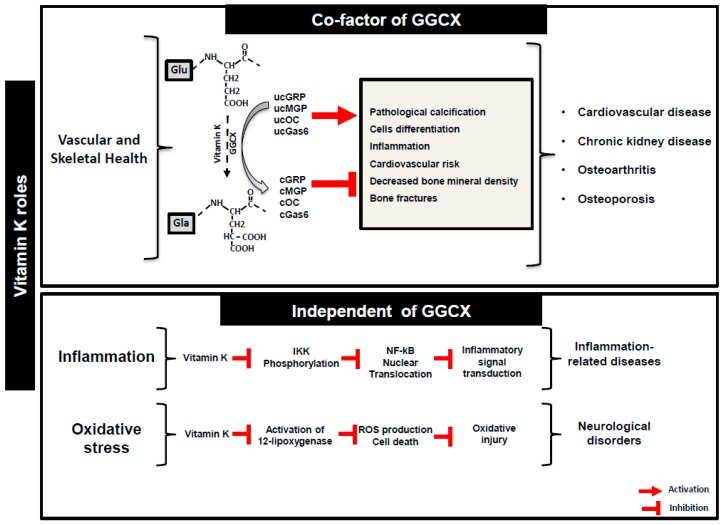
Vitamin K roles in health and disease. Vitamin K function as a co-factor for γ-glutamyl carboxylase enzyme (GGCX) essential for the γ-carboxylation of target vitamin K-dependent proteins (VKDPs), but also independent of GGCX as an anti-inflammatory and antioxidant agent. Uncarboxylated (uc) protein forms of VKDPs, such as GRP, MGP, OC and Gas6, have been implicated in several pathological processes occurring in multiple age-related diseases, while the γ-carboxylated form of these proteins (c) are known to have a health protective role. Glu, glutamic acid; Gla, γ-carboxyglutamic acid; IKK, nuclear factor κB kinase; NF-κB, nuclear factor κB.

Vitamin K is a family of naphthoquinone compounds comprising K1 (phylloquinone) and several forms of K2 (MKs, menaquinones). Phylloquinone is synthesized exclusively by plants, algae, and some species of cyanobacteria. Menaquinones are mainly produced by obligate and facultative anaerobic bacteria [1], with long side chains (MK7 to MK13) with the exception of MK4, that is not a common product of bacterial synthesis, but is considered to be of animal origin based on its tissue-specific conversion from phylloquinone [2]. In terms of structure, both forms are characterized by the presence of a common polar, hydrophilic 2-methlyl-1,4-naphtoquinone ring (menadione, vitamin K3) and a hydrophobic polyisoprenoid side chain that can vary in length and bond saturation. Vitamin K1 has a phytyl side chain containing only one unsaturated bond. Vitamin K2 family (MK-n) is divided in a series of isoprenologs where n is the number of prenyl units in the isoprenoid side chain. Vitamin K has been first reported for its role as a cofactor for the microsomal enzyme γ-glutamyl carboxylase (GGCX) ensuring the correct function of vitamin K-dependent hepatic clotting factors. The posttranslational protein modification that converts specific glutamic acid residues (Glu) into γ-carboxyglutamate (Gla), is crucial for the function of vitamin K-dependent proteins (VKDPs), also known as Gla proteins. However, the health benefits of this micronutrient were shown to extend beyond coagulation, and to have an extra-hepatic role ensuring the correct function of several extra-hepatic VKDPs. These have been described to play different roles in both physiological and pathological processes such as tissue mineralization, neuroprotection, energy metabolism, inflammation and cellular growth and survival. Also, the potential use of vitamin K2 in the prevention and clinical treatment of cancer was recently reviewed [3]. This multifunctional vitamin has been implicated in chronic age-related diseases such as cardiovascular diseases, osteoporosis and osteoarthritis [4]. More recently, a novel role has been disclosed for vitamin K as an antioxidant and anti-inflammatory, independent of its activity as a cofactor for GGCX. The function of VKDPs associated to these emerging functions of vitamin K is now starting to be explored. Vitamin K status has been associated with lower concentrations of inflammatory markers in vivo [5], and proposed to exert an anti-inflammatory role by suppressing nuclear factor κB (NF-κB) signal transduction [6,7]. Also, a protective effect against oxidative stress was suggested for this micronutrient through blocking of reactive oxygen species (ROS) generation [8] (Figure 1).

Several chronic inflammatory and mineralization-related diseases have been associated with vitamin K deficiency, namely cardiovascular disease (CVD), chronic kidney disease (CKD) [9], osteoarthritis (OA) [10] and osteoporosis. Since these are highly prevalent age-related health conditions, and both inflammation and pathological mineralization are associated with the aging process, a new concept of vitamin K involvement in aging and ’inflammaging’ is arising. Given the growing amount of data regarding vitamin K and VKDPs functionality and beneficial effects, it is not surprising that it could be considered a super micronutrient used in clinical practice as a health supplement in the near future. In this work, we thoroughly review available data regarding clinical studies involving the evaluation of vitamin K in skeletal and cardiovascular health, highlighting that definitive conclusions are still warranted to clearly impose vitamin K supplementation as a health benefit. We also address the role of vitamin K in aging conditions such as functional decline, disability, sarcopenia and frailty, affecting the elderly in an aging society. Simultaneously, we explore the protective role of vitamin K in aging and age-related diseases, through its involvement as a modulator in the interplay between pathological calcification and inflammation processes.

## 2. Vitamin K and Health: Evidences from Clinical Studies 

Besides its role in maintaining normal blood coagulation, vitamin K and VKDPs play an important role in human bone metabolism and soft tissue physiology, and are mechanistically associated with the onset and progression of several age-related chronic diseases [10,11,12,13].

A chronic low-grade pro-inflammatory state and calcification of connective tissues have been implicated in age-related diseases such as cardiovascular disease (CVD) and osteoarthritis (OA). Both conditions are highly prevalent in the aging population and, as the number of patients will continue to rise, this constitutes a major worldwide challenge for our health system. Currently there are no effective treatments available to prevent, stop, or even restrain CVD and OA progression. Treatments are targeted to relieve symptoms, prevent complications and reduce the risk of recurrence or worsening. Inflammation and pathological calcification not only play a key role in CVD and OA, but are also involved in a complex interplay, ultimately leading to disease progression. Understanding the underlying molecular mechanisms driving these processes will lead to the discovery of new biomarkers and therapeutic strategies for these age-associated diseases. Research efforts on the role of vitamin K and VKDPs in diseases of aging have been mainly focused on the action of matrix Gla protein (MGP) and osteocalcin (OC). Gla-rich protein (GRP) is a VKDP, and was recently shown to have a dual capacity to act as an anti-inflammatory agent and an inhibitor of pathological calcification in the articular and vascular system. Novel and more efficient diagnostic and therapeutic options are urgently needed to lower the burden and the associated health care costs of CVD and OA in the aging population.

Several population-based studies and randomized trials focusing on the association of different forms of vitamin K and CVD and OA outcomes, are available or presently ongoing. Furthermore, an association between CVD and OA has been highlighted in recent years. In a cohort study involving 2156 participants with hip and/or knee OA aged over 55 years, OA disability was significantly associated with mortality and serious CVD events [14]. A previous population-based cohort study, involving 1163 OA patient follow-up for a median of 14 years, also reported that increased mortality in people with OA, was largely due to CVD causes [15].

### 2.1. Circulating Biomarkers to Evaluate Vitamin K Status 

Considering the natural vitamers of vitamin K, K1 and K2, vitamin K status is dependent on both the dietetic intake and intestinal bacterial synthesis. According to the Panel on Dietetic Products, Nutrition and Allergies (NDA) of the European Food Safety Authority, none of the available biomarkers of vitamin K intake or status are considered suitable to derive dietary reference values for vitamin K. The assessment of vitamin K nutritional status in population and clinical-based studies is still of crucial importance to unveil the role of vitamin K in human health and age-related chronic diseases [16]. In terms of vitamin K status, the literature unanimously considers that there is no “gold standard” biomarker to measure vitamin K sufficiency or deficiency. Instead, vitamin K status has been evaluated using multiple biomarkers, each reflecting a different aspect of vitamin K intake, storage, bioavailability and function. Common direct methods include the quantification of urinary vitamin K1 metabolites [17] and of circulating forms of vitamin K1 and K2 [18]. According with a recent review [19], most of the available data on circulating vitamin K is based on direct measurements of circulating K1, which is still considered by many researchers a global indicator of vitamin K status. One limitation of this measurement is that it only reflects vitamin K short-time intakes, due to the short half-life of the K1 vitamer [20]. Most of these available studies, using direct quantification methods, are focused on the assessment of dietary vitamin K levels, instead of its association with health and pathologic conditions. Still, limited information is available in the literature for plasma menaquinones, mostly because (except for MK4) their concentrations are much lower and generally not detectable in circulation, unless large quantities are consumed [19]. 

On the other hand, a considerable number of clinical studies describe the use of indirect methods, measuring circulating markers representing uncarboxylated VKDPs, namely OC and MGP, to reflect extra-hepatic vitamin K status, or study the effect of vitamin K supplementation, and/or its association with a particular health condition. The choice of the appropriate biomarker for a specific clinical study needs to be addressed based on the research question to be explored, together with the available scientific evidences sustaining its association with the particular health condition. Several assays measuring different forms of OC and MGP fractions in circulation have been developed [21,22], and validated in clinical assays for their ability to reflect vitamin K status [23]. Clinical studies focused on CVD have chosen to use circulating levels of desphospho-uncarboxylated matrix gla protein (dp-ucMGP), since this was found to be the best biomarker to reflect vitamin K status [23] and vascular calcification (VC). Indeed, several randomized controlled studies have shown the effect of vitamin K supplementation in lowering circulating levels of dp-ucMGP [24,25,26]. In a randomised, double-blind, placebo-controlled trial to investigate the effect of MK-7 supplementation, the authors found a dose-dependent decrease of uncarboxylated MGP concentrations [27], supporting the choice of dp-ucMGP as a non-invasive marker of vitamin K status. Also, studies in healthy subjects to evaluate changes in systemic vitamin K status showed a decrease in dp-ucMGP levels, resulting from vitamin K supplementation, and an increase following treatment with a vitamin K antagonist (VKA, coumarin derivatives such as warfarin) [23]. An observational cohort study, involving 160 prevalent haemodialysis (HD) patients [28], and a relatively small study enrolling a total of 60 kidney transplants recipients [25], have both confirmed that VKA therapy was associated with higher dp-ucMGP levels. Interestingly, a study measuring dp-ucMGP in seven HD patients switching from VKA to fondaparinux (an indirect factor Xa inhibitor), showed that stopping VKA treatment is associated with a rapid reduction of dp-ucMGP concentrations, to levels observed in HD patients not treated by VKA [29]. 

Osteocalcin is a VKDP synthetized in bone and circulating uncarboxylated form (ucOC) has been described to reflect low vitamin K status [30]. In clinical studies addressing bone health, ucOC is usually selected as a biomarker of vitamin K status [31,32,33]. Also, circulating undercarboxylated prothrombin, known as PIVKA-II (protein induced by vitamin K absence or antagonism–II), is a functional marker to access vitamin K status in response to vitamin K restriction, such as to VKAs [34]. Nevertheless, currently available PIVKA-II commercial assays have been reported as lacking sensitivity to detect variation in vitamin status associated with healthy populations [19]. 

### 2.2. Vitamin K Status and Skeletal Health 

Low vitamin K status has been associated with multiple co-morbidities, functional decline and disability in older adults, especially in those with associated age-related diseases such as OA and osteoporosis [11,35]. 

#### 2.2.1. Functional Decline and Disability

In a cross-sectional and longitudinal analysis enrolling 1089 community-dwelling older adults from the Health ABC Study, a lower vitamin K status measured by plasma K1 levels and dp-ucMGP, was associated with worse lower-extremity function over 4–5 years of follow-up [36]. In the same analysis, dp-ucMGP was not consistently associated with lower-extremity function, suggesting a direct effect of vitamin K on skeletal muscle, independent of its function as a co-factor of GGCX. In a following study of the same Health ABC cohort, the authors evaluated the association between vitamin K status and incident mobility limitation and disability. Although no underlying mechanism is proposed, the results suggest the involvement of vitamin K in the disabling process associated with aging. Older adults with lower levels of plasma K1 were found to be more prone to develop mobility limitation and disability, compared to those with higher circulating levels [37]. In a prospective study involving 13 years of follow-up of 644 community-dwelling adults from the Longitudinal Aging Study Amsterdam (LASA) cohort, low baseline vitamin K status (high levels of circulating dp-ucMGP) was associated with a higher frailty index score in older aging people [38]. A previous longitudinal analysis in this LASA cohort (13 years follow-up), showed that a lower status of vitamin K was associated with lower grip strength and calf circumference, reinforcing the association of the physical component in the development of frailty. This highlights the importance of vitamin K to prevent and slow down the development of frailty in an aging population and precludes its beneficial use as a nutritional supplement.

#### 2.2.2. Osteoporosis

The effect of vitamin K supplementation on bone metabolism has been evaluated in several randomized clinical trials. Most of these studies were conducted with postmenopausal women with osteoporosis, and generally concluded that both vitamin K1 and K2 had a protective effect on bone health, by increasing bone strength and reducing bone turnover, while decreasing the risk of fractures [35,39,40,41,42,43]. Nevertheless, some inconsistencies are still found in the literature. A study performed within the Danish Osteoporosis Prevention Study (DOPS) including a cohort of 2016 perimenopausal women, concluded that vitamin K1 supplementation had no effect in bone mineral density and fracture risk [44]. In another study performed in the community-based Hordaland Health Study, a low intake of vitamin K1 was associated with an increased risk of fractures, while K2 showed no association [45]. Regarding this issue, a study comparing absorption and efficacy of K1 and MK7 showed that both forms were well absorbed but MK7 was able to induce a more complete carboxylation of OC in bone [46]. In fact, high doses of vitamin K2 have been shown to be effective in the prevention and treatment of osteoporosis, although most of these studies were initially done in Japan [41,47,48,49,50]. A randomized clinical trial comprising a total of 4378 women with osteoporosis, concluded that the beneficial effect of supplementation with 45 mg/day menatetrenone (MK4) was only demonstrated in postmenopausal women with advanced osteoporosis [51]. A meta-analysis including 19 randomized controlled trials and encompassing 6759 participants was conducted to estimate the effect of vitamin K2 for the prevention and treatment of osteoporosis in postmenopausal women [52]. The authors conclude that K2 treatment was able to improve vertebral bone mineral density (BMD) and reduce fracture risk in osteoporotic postmenopausal women, although no effect was seen in postmenopausal women without osteoporosis. A systematic review and a meta-analysis including 36 randomized controlled trials with a total of 11,112 participants addressing the effect of vitamin K supplementation on BMD and fractures in adults was recently published. Most of the trials included in the analysis used the MK4 form of vitamin K2 (45 mg/day), although many also used vitamin K1 and some MK7 at various dosages [53]. From this study, vitamin K supplementation appears to have no effect on vertebral fracture outcomes and little effect on BMD, for postmenopausal and osteoporotic patients. 

In fact, whether the effect of vitamin K supplementation, inducing a decrease in circulating ucOC, leads to improvement of bone quality and to reductions in BDM loss and fractures in postmenopausal healthy women, remains a matter of divergence and debate in the literature [54]. This could be due to limitations such as small study samples, which difficult the confirmation of the role of vitamin K in bone pathology. In a large-scale interventional study, the Japanese Osteoporosis Intervention Trial-03 (JOINT-03), a total of 1983 osteoporotic women (mean age 75.3 years) were randomly allocated into two groups, one including 931 patients treated with vitamin K2 (45 mg/day) and risedronate (2.5 mg/day) and the other (*n* = 943) taking risedronate alone (2.5 mg/day). In both groups, the incidence of fractures, changes in BMD, height and ucOC concentration, was evaluated over a 2-year follow-up. The authors concluded that, in terms of fracture prevention, there was no difference between both treatments [55]. Also, given the variability of vitamin K2 supplementation doses included in the different studies, and the different conclusions, it is reasonable to suggest that different formulations of vitamin K appear to have different levels of potency, and that higher doses might be necessary to fully carboxylate OC. Recently, an open-labelled prospective cohort study evaluated the effect of escalating doses of MK4 on the improvement in OC carboxylation. The study was conducted in postmenopausal women with osteoporotic fractures, and showed that supplementation with 5 or 45 mg/day of MK-4 reduced ucOC to levels usually reported in young and healthy adults. The authors also concluded that higher doses of MK4 were more effective, with no major side effects [56]. 

#### 2.2.3. Osteoarthritis (OA) and Rheumatoid Arthritis (RA)

Evidences from preclinical studies have suggested a role for vitamin K in OA prevention. Clinical studies on the effects of vitamin K on OA are very limited and most addressing only vitamin K1. The first indication of an association between low plasma levels of vitamin K and increased prevalence of knee and hand OA features came from an observational analysis involving 672 participants from the Framingham Offspring cohort [57]. The same results were reported in another observational study using a survey information on dietary intake obtained from 719 subjects of the elderly Japanese population [58]. A longitudinal analysis with 1180 subjects from the Multicenter Osteoarthritis (MOST) Study concluded that subclinical levels of plasma K1 were associated with an increased risk of OA development [10]. Consistent with these results, another longitudinal study involving participants from the Health ABC Study [59] showed that subjects with very low plasma K1 were more likely to have articular cartilage loss over the three years follow-up [11].

VKDPs, namely OC, MGP and more recently GRP, have been shown to be associated with OA. A single-arm clinical study involving 25 Japanese patients with bilateral knee OA determined ucOC serum levels, and suggested that ucOC could be a useful marker for evaluating the pathophysiological condition of knee OA [60]. Results from in vitro and ex vivo experiments have shown that uncarboxylated MGP (ucMGP) was associated with increased mineralization in OA cartilage, suggesting that vitamin K deficiency may contribute to abnormal mineralization in OA [61]. Data from an observational cross-sectional study, involving 26 arthritis patients and 30 controls, showed that the ratio of synovial to serum ucMGP was significantly higher in inflammatory arthritis versus non-inflammatory arthritis. However, although higher levels of serum ucMGP were found in arthritis patients when compared with controls, the results did not reach statistical significance [62]. 

Also, 791 knee OA participants were drawn from the Health ABC Study for a cross-sectional analysis, and 523 for a 3-year longitudinal observational study, to investigate the association of K1 with structural features of knee OA in older community-dwelling adults. Individuals with elevated levels of dp-ucMGP at baseline were found to be more likely to have osteophytes, bone marrow lesions, subarticular cysts, and meniscal damage. Participants with very low plasma K1 had a 1.7- and 2.6-fold higher odds of worsening cartilage and meniscal damage over 3 years, although dp-ucMGP was not associated with progression of any OA features [11]. Based on these findings, the authors suggested the possible involvement of vitamin K in OA independent of its function in MGP carboxylation. 

Interventional studies assessing the effects of vitamin K1 supplementation in OA and RA are available. A 3-year randomized controlled trial, involving 378 participants assessing the effect of vitamin K1 supplementation (500 μg/day) on radiographic hand OA in older adults, failed to find a beneficial effect in hand OA progression as compared to the placebo group [63]. Recently, a small double-blind placebo-controlled trial, enrolling 58 patients with rheumatoid arthritis and including vitamin K1 supplemented (10 mg/day) and placebo groups, concluded that after the 8 weeks follow-up, there were no significant differences between the two groups regarding blood biomarkers of inflammation or disease severity [64]. 

In fact, mineralization and inflammatory processes have been shown to be intimately linked in a feed-back loop that will ultimately lead to OA development and progression [65,66,67]. In addition to MGP and OC, GRP was recently associated with the OA pathophysiology. Evidences from pre-clinical studies have shown a predominance of the undercarboxylated forms of GRP in cartilage and synovial membrane from OA patients [68,69]. GRP was suggested as a novel factor linking the two main pathological processes associated with OA, mineralization and inflammation. 

In a study enrolling 158 female patients suffering from rheumatoid arthritis (RA) [70], vitamin K2 (MK4) supplementation was shown to be associated with a reduction of inflammation and disease activity. A cross-sectional prospective study enrolling 84 RA patients was performed in order to elucidate the therapeutic role of MK7. After 3 months, the supplemented group showed a significant reduction in markers of inflammatory status and a reduction in disease activity score [71].

Of note, several in vitro studies have been supporting a novel function for vitamin K, as an anti-inflammatory agent independently of its role as a cofactor of GGCX, which might also be an alternate pathway influencing joint health (further detailed below) [7,72,73]. 

Although no definitive information on cause-and-effect relation can be derived from the available studies, overall, they strongly indicate a beneficial role of vitamin K in bone and cartilage health, and further suggest a protective effect of vitamin K supplementation in OA. Further and larger sample clinical interventional studies, on the effect of vitamin K in OA prevention, are needed to evaluate the efficacy of the different vitamin K forms as therapeutic agents and to unveil their role as modulators in the interplay between mineralization and inflammation, driving the disease progress.

### 2.3. Vitamin K Status and Cardiovascular Diseases (CVD) 

Several lines of evidence from preclinical studies have established the involvement, at tissue and systemic level of vitamin K in VC, mainly through carboxylation of two vitamin K-dependent proteins: MGP and GRP. In the last 10 years, a growing number of observational and longitudinal clinical studies on the relation between vitamin K status and cardiovascular health have arisen. Dietary vitamin K is believed to decrease the risk of CVD, but inconsistent results are reported. This may be due to different effects of vitamin K vitamers (K1 and K2), although most studies do not include both. In fact, cross-sectional studies reported an inverse association between K2 intake and incidence of coronary heart disease (CHD) and coronary calcification [74,75], consistent with a protective role of dietary vitamin K against the incidence of cardiovascular events. These studies included an investigation of vitamin K1 and several subtypes of K2. In an observational cross-sectional study enrolling 564 Dutch women, high intake of K2 was associated with decreased coronary calcification, but K1 intake was not [74]. Previously, the Rotherham Study also investigated both K1 and K2 intake, but relations of the different K2 subtypes with coronary calcification were not addressed. In this study, a protective effect of dietary K2 intake against CHD in older men and women was suggested, with no consistent association of K1 intake with CHD, mortality or aortic calcification [75]. Further, a prospective study using data from a cohort of 16,057 elderly women followed for 8.1 years, showed that a higher dietary vitamin K2 intake was significantly associated with a lower incidence of CHD, and mainly driven by MK7, MK8 and MK9 subtypes [76]. 

In the majority of the reported studies, the dp-ucMGP is used to assess the cross-sectional relation between circulating vitamin K status and CVD, since it has been considered as the unique circulating MGP form reflecting, at extra-hepatic tissue level, vitamin K status and function [27]. As recently reviewed [77,78], several clinical observational studies have shown an association between increased levels of dp-ucMGP with VC and CVD-related outcomes. These studies were mainly performed in specific high-risk population groups, such as CKD patients [79], diabetic and hypertension patients [80], or long-term warfarin anticoagulant therapy users [81]. Overall, a growing amount of evidence from clinical observational studies indicates an association between vitamin K status and higher levels of circulating dp-ucMGP, with a higher cardiovascular risk, including higher coronary artery calcification (CAC), increased peripheral arterial calcification, higher carotid-femoral pulse wave velocity and increased arterial stiffness. Two cross-sectional studies have shown that subjects with the highest dp-ucMGP had a positive association with increased arterial stiffness, which is also an independent risk factor for CVD [82,83]. Also, a longitudinal study enrolling 577 community dwelling older participants in the Longitudinal Aging Study Amsterdam (LASA), without prevalent CVD at baseline, showed that after a mean follow-up of 5.6 ± 1.2 years, higher plasma dp-ucMGP was correlated with a significant higher CVD risk [84]. A prospective longitudinal cohort study including 635 participants drawn from the Health ABC Study [59] was performed to determine the association between plasma K1 and dp-ucMGP with the risk of CVD over a period of 12 years [12]. The results from this study showed that low plasma K1 was a risk factor for CVD in older individuals treated for hypertension, but no association was demonstrated in the non-treated group. In a recent meta-analysis, including a total of 11 prospective cohort studies involving 33,289 patients, the association between dp-ucMGP and the risk of cardiovascular events and mortality was assessed. The results indicated that higher circulating levels of dp-ucMGP, indicative of vitamin K insufficiency, were significantly associated with a higher CVD and total mortality risk. As a conclusion, the authors further suggest that dp-ucMGP may serve as an independent risk of mortality, although emphasizing that more and larger-populations studies are needed to confirm this association [13]. However, in a post-menopausal cross-sectional study, where dp-ucMGP was reduced by vitamin K1 supplementation, changes in dp-ucMGP did not correlate with CAC [85]. Also, in a general healthy population a case-cohort study involving 11 years of follow-up, showed that high dp-ucMGP levels, reflecting poor vitamin K status, were not associated with an increase in CHD or stroke risk [86]. Interestingly, a prospective cohort in healthy postmenopausal women showed that higher circulating levels of K1 were unexpectedly associated with increased prevalence of CAC. The authors suggested that the K1 status obtained from dietary intake in this observational study, might not be able to sustain a protective effect on CAC [87].

In a randomized trial (*n* = 244, mean age 59.5 ± 3.3 years), 3-years of supplementation with a nutritional dose of natural MK7 (180 μg/day) was shown to decrease arterial stiffness in healthy postmenopausal women, especially in a subgroup of women with high local carotid artery stiffening [88]. A low vitamin K status in CKD patients has been associated with a high prevalence of VC and cardiovascular mortality [9,89]. In 2014, a prospective, randomized, single-blinded intervention study was performed, including 200 chronic haemodialysis patients, to evaluate the effect of high-doses of MK7 supplementation [24]. This study, showing a dose-dependent decrease in dp-ucMGP by MK7 supplementation, was further confirmed in a more recent clinical intervention trial with MK7, including 50 haemodialysis patients [90]. The conclusions will be useful for further studies on larger interventional trials, addressing the effect of vitamin K supplementation on CVD progression and mortality in the haemodialysis population. 

Several clinical studies are currently ongoing to evaluate the efficacy of vitamin K supplementation in CVD progression. The VitaVasK is a prospective, randomized study that will include 348 haemodialysis patients, and aims to study the effect of vitamin K1 in reducing VC progression in CKD patients [91]. The VitaK-CAC trial is a double-blind, randomized, placebo-controlled trial, currently investigating the effect of MK7 supplementation in patients with coronary artery disease, and also aiming to evaluate the effect on disease progression [92]. 

So far, the conclusions drawn from clinical studies are still quite limited by the small sample number and the substantial associated heterogeneity. More robust clinical trials, including a larger number of patients, addressing the effect of the different forms of vitamin K supplementation to improve cardiovascular calcification/stiffness, thereby reducing the risk of cardiovascular events and promoting long-term cardiovascular health, are urgently needed.

In fact, some answers are soon to be expected from ongoing clinical trials. The Aortic Valve Decalcification trial (AVADEC) is a randomised, placebo-controlled study that will examine the effect of MK-7 on progression of aortic valve calcification (AVC) for a period of at least 24 months [93]. The study is planned to include a total of 400 patients with an AVC score above 300 but without aortic valve stenosis. Half of the patients will be randomized to placebo treatment, and the other half for supplementation with MK-7. If positive effects are shown from this interventional study, this could represent new treatment options to prevent progression of AVC.

Of note, a recent observational prospective study has highlighted the potential role of circulating GRP as a novel biomarker of VC. In this study enrolling 80 type 2 diabetic patients with CKD stages 2–4, total GRP serum levels were found to be associated with deterioration of renal function and to be an independent risk factor of VC in this population [94]. In this study circulating GRP was measured using a recently published sandwich ELISA system, able to detect total GRP, independently of its γ-carboxylation state [95]. Although this was the first study exploring the relation between GRP circulating levels and VC at clinical level, the results showed that decreased serum GRP could be a clinical predictor of pathological calcification in CKD, with promising and broader applications as a new marker for cardiovascular calcifications. 

## 3. Vitamin K in the Interplay between Calcification and Inflammation in Age-Related Diseases. Evidences from Pre-Clinical and Clinical Studies 

Aging is a well-established major risk factor for all chronic diseases and syndromes associated with old age. Vitamin K has been suggested to play a protective role in aging and age-related diseases. Highly prevalent diseases such as CVD and OA still represent a major health challenge in our aging society worldwide. CVD and OA are chronic low-grade inflammatory diseases that have been associated with increased risk of mobility disability and frailty, in the elderly. Also, vitamin K has been implicated in chronic diseases that lead to mobility disability and frailty. Although a beneficial effect of vitamin K has been suggested to prevent and ameliorate these age-related conditions, its underlying mechanism of action is not yet known. 

**Figure 2:**
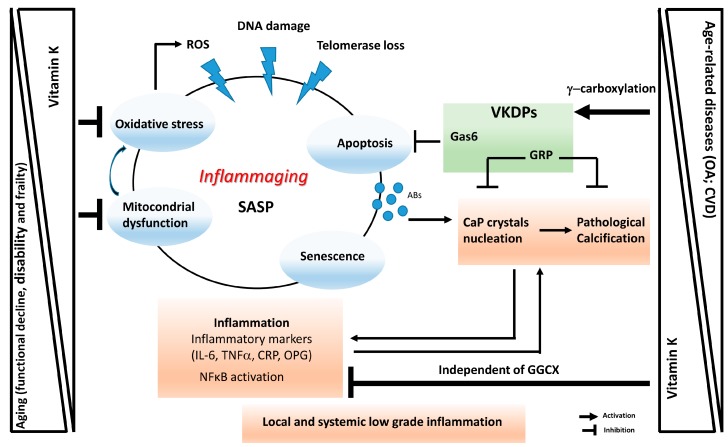
Schematic representation of vitamin K involvement in ’inflammaging’ and age-related diseases. ’Inflammaging’ and its associated senescence-associated secretory phenotype (SASP) can be triggered by stress conditions such as reactive oxygen species (ROS), DNA damage and telomerase loss, resulting in increased oxidative stress, mitochondrial dysfunction, cell senescence and apoptosis. All these processes contribute to an increased low-grade inflammatory status at tissue and systemic levels. Decreased vitamin K levels have been associated to increased aging processes and age-related disorders, by interfering with the γ-carboxylation of VKDPs such as Gas6 and GRP involved in apoptosis and pathological calcification, and by modulating inflammation, oxidative stress and mitochondrial dysfunction independently of its activity as co-factor for γ-glutamyl carboxylase (GGCX). Abs, apoptotic bodies; OA, osteoarthritis; CVD, cardiovascular diseases.

The development of a pro-inflammatory status, characterized by high levels of inflammatory markers present at local and systemic levels, is a common characteristic associated with aging and age-related diseases such as CVD, type 2 diabetes, CKD, cancer, osteoporosis, OA, frailty, sarcopenia and several neurological disorders. The human aging process characterized by a chronic, low grade proinflammatory state was coined in 2000 as ‘inflammaging’ [96]. More recently, a related concept termed ‘garb-ageing’ was introduced. This concept emphasizes the decline in cell autophagy, that becomes impaired with aging. ‘Garb-ageing’ refers to the accumulation of “molecular garbage” consisting of endogenous/self, misplaced/altered macromolecules resulting from damaged/dead cells or organelles (cell debris), ultimately leading to the continuous activation of inflammasome in macrophages [97]. Multiple stressors and damaging agents such as ROS, DNA damage agents and telomere loss, can trigger cell senescence, which is characterized by a quiescent cell state with a pro-inflammatory phenotype [98]. This altered phenotype is known as senescence-associated secretory phenotype (SASP) and involves the production of high levels of pro-inflammatory cytokines, chemokines, growth factors and matrix metalloproteinases (MMPs). With aging or in a pathologic context, senescent cells are known to accumulate in tissues, promoting local and systemic inflammation [96,98]. The SASP has been recently described to promote age-related pathologies such as OA and CVD [99,100,101], and to be a significant risk factor for morbidity and mortality in the elderly (reviewed in [99]) (Figure 2). 

Mitochondrial function has been reported to be reduced with aging in terms of adenosine triphosphate (ATP) production and respiratory chain activity [102,103]. Oxidative stress induces damage to mitochondrial DNA resulting in altered abundance and function of mitochondrial proteins. A considerable body of literature has reported that an increase in mitochondrial dysfunction can lead to the establishment of a low-level chronic and systemic inflammation status, resulting in increased oxidative stress, which promotes a vicious inflammatory cycle that modulates most chronic age-related diseases (reviewed in [104,105]). In this context of mitochondrial pathology, there are still no studies available in mammalian cells addressing a direct effect of vitamin K. Nevertheless, a study in Drosophila proposes vitamin K2 as a promising compound to treat mitochondrial pathology. In this study, MK4 can act as a mitochondrial electron carrier, like ubiquinone, facilitating ATP production, rescuing mitochondrial defects and restoring its function [106]. 

All these structural and genomic changes involving several factors such as cell senescence, oxidative stress and inflammatory mediators, lead to an increase in apoptotic processes which have also an important role in several chronic age-related diseases. Apoptosis has been for long described to be of pathophysiological significance in CVD and OA [107] (Figure 2). Results from in vitro and ex vivo studies have shown that articular chondrocytes-derived apoptotic bodies contribute to the pathologic cartilage calcification and extracellular matrix degradation, observed in OA and aging [108,109]. Apoptotic bodies were shown to be able to perform the same role as matrix vesicles, promoting the formation of calcium phosphate (CaP) crystals. The same is reported in vascular smooth muscle cells (VSMCs) where apoptotic bodies are known to act as nucleating structures for CaP formation [110]. CaP crystals also have a role at systemic level, as part of circulating calciprotein particles (CPPs) and mineralization-competent extracellular vesicles (EVs). Several studies have demonstrated the toxicity of CaP nanocrystals and their ability to promote pro-inflammatory reactions, which, in turn, induce pro-calcific responses in a cycle were mineralization triggers inflammation and vice-versa. Mineralization and inflammation have been shown to play a key role in the pathophysiology of CVD and OA. The complex crosstalk between both processes leads to an amplified response that ultimately contribute to disease progression. Nowadays, it‘s currently accepted that highly complex mechanisms regulate the formation, maturation and consequent pathogenicity of CaP nucleation sites. At the same time, soft tissue mineralization is an active and naturally occurring multifactorial process that must be actively inhibited [111]. This emphasises the concept that inhibition of CaP crystal formation and maturation should bring important results in the prevention and management of these age-related diseases.

In the context of apoptosis, the VKDP Gas6 has been shown to exert anti-apoptotic effects on endothelial and VSMCs [112,113,114], by binding and activating the receptor tyrosine kinase Axl [112,115,116]. More recently, an in vitro study using primary cultured rat VSMCs, showed that treatment with vitamin K2 was able to inhibit VSMC calcification by preventing apoptosis through restoration of the Gas6/Axl/Akt pathway [117], further indicating a potential contribution of vitamin K for the treatment of CVD.

The anti-inflammatory activity of vitamin K has been demonstrated in several in vitro and animal studies. In a study using human primary cultured fibroblasts challenged with the lipopolysaccharide (LPS) endotoxin, MK4 was found to suppress IL-6 production, with higher efficiency than vitamin K1. The same results were obtained in THP-1 human macrophages and murine RAW264.7. In this study, all vitamin K forms (K1, MK3, MK4 and MK7) were found to supress LPS-induced expression of IL-6 similarly. Furthermore, the anti-inflammatory effect of vitamin K was shown to be independent of its Gla formation activity. After treatment of the cells with LPS, MK4 reduced the activation of NF-κB, and inhibited IKKα/β phosphorylation [7]. Also, vitamin K1 supplementation was shown to supress the inflammation state induced by LPS in rats [72]. Of note, several clinical studies have demonstrated that vitamin K status in humans is inversely correlated with circulating inflammatory markers. In a cross-sectional study enrolling 379 healthy men and postmenopausal women (mean age 68 ± 6 years; 58.5% women), plasma vitamin K1 was found inversely correlated with IL-6 and C-reactive protein (CRP), considered a marker of systemic inflammation, although no association was found for serum %ucOC. Nevertheless, no differences were noted in the 3-year follow-up of patients receiving vitamin K1 supplementation, relatively to the concentrations of inflammatory biomarkers. The lack of effect of vitamin K1 supplementation on circulating cytokines obtained in this placebo-controlled trial, was suggested to be due to the healthy status of this cohort [118]. In another cross-sectional community based-cohort analysis, enrolling participants from the Framingham Offspring Study (*n* = 1381; mean age 59 ± 8 years), circulating levels of vitamin K1 and K1 intake, were both found inversely associated with IL-6 and osteoprotegerin inflammatory markers [5]. Interestingly, %ucOC was not associated with overall inflammation, supporting the previous in vitro evidences showing that vitamin K modulates inflammation by a mechanism independent of its role in Gla formation. 

Consistently with previous cross-sectional analyses in Caucasian adults [118], an observational study performed in 662 community-dwelling adults (mean age 62 ± 10 years) from the Multi-Ethnic Study of Atherosclerosis (MESA), without clinical apparent CVD, showed that serum K1 was significantly and inversely associated with several circulating inflammatory markers namely IL-6, CRP and intercellular adhesion molecule 1 (ICAM-1) [119]. In a cohort analysis of the Health ABC including 1163 older adults (mean age 74 ± 3 years), those with lower circulating levels of K1 at baseline, had also higher circulating IL-6 levels, further supporting the potential anti-inflammatory effect of vitamin K at systemic level [12].

A double-blind randomized clinical trial, involving 244 healthy postmenopausal women, receiving either placebo or MK7 for 3 years, showed that MK7 supplementation did not influence circulating inflammatory markers. Nevertheless, IL-6 and high-sensitivity C-reactive protein (hsCRP) correlated with vascular vitamin K status (measured by dp-ucMGP) and arterial stiffness at baseline [88]. Contrarily, in a cross-sectional prospective study involving RA patients on MK4 supplementation for 3 months, a significant reduction in inflammatory markers was observed [70]. Overall, the number of clinical studies aiming to evaluate the benefits of the use of vitamin K supplementation in modulating inflammation is still limited. From the available results we suggest that patients with inflammatory diseases, rather than healthy elderly, would be a more adequate population to study the efficacy of vitamin K supplementation on the inflammatory state. This subject clearly merits further investigation.

As a final note on the relation between vitamin K and inflammation, the potential role of GRP should be further explored at clinical level. Although the association of circulating total GRP [95], with vascular calcification has been recently shown in a small observational prospective study in type 2 diabetic patients with CKD, no indications were yet disclosed relatively to the association with inflammatory markers [94]. Several in vitro and ex vivo evidences have demonstrated that this VKDP has a dual capacity to act as an inhibitor of VC and as an anti-inflammatory [69,120]. This role was reinforced when supplementation of CPPs isolated from CKD stage 5 patients with γ-carboxylated GRP (cGRP), were able to rescue the calcification/osteogenic differentiation and inflammatory status induced in VSMCs [95]. 

Recently, low vitamin K status has been associated with multiple co-morbidities, functional decline, disability and frailty in older adults [37,121], especially in those with associated OA and CVD [11,122]. In addition, inflammation accelerates ageing in individuals with multimorbidity, and further promotes the functional limitation and disability in the elderly [123]. Several cross-sectional studies describe an association between inflammation and frailty in older adults [124,125,126]. High levels of circulating pro-inflammatory cytokines, such as CRP, tumor necrosis factor alpha (TNF-α), and IL-6 were shown to promote sarcopenia, a syndrome related with frailty and characterized by muscle breakdown and increased risk of muscle mass and strength loss [126,127]. In a population consisting of 1323 older community-dwelling adults (mean age 74.6 ± 2.8 years) drawn from the Health ABC cohort study, plasma vitamin K1 was inversely associated with IL-6 and knee pain. Also, dp-ucMGP was found to be positively associated with IL-6 and mobility disability in this cohort [36]. 

Importantly, vitamin K1 and K2 have been shown to have antioxidant properties in cultured neurons and oligodendrocytes, independently of its role in the carboxylation of VKDPs [8,128]. This study showed that cell death, caused by oxidative stress was able to be prevented, by inhibiting activation of 12-lipoxygenase, which could represent an alternative anti-inflammatory mechanism associated with vitamin K. In vertebrates, vitamin K epoxide reductase complex subunit 1 (VKORC1)-like 1 (VKORC1L1), the paralogous enzyme of the VKORC1, is described to play a fundamental role in intracellular antioxidation, by protecting cells from oxidative damage of membrane intrinsic proteins [129]. Furthermore, two kinetic studies showed that vitamin K1-hydroquinone is a strong antioxidant, 10–100 times more potent when compared with other biological radical scavengers such as vitamin E (α-tocopherol), and ubihydroquinone-10 [130], suggesting a role in preventing lipid peroxidation in biological systems. In another study, aiming to test the effect of the vitamin K cycle on lipid peroxidation in rat liver microsomes, vitamin K1 and K2 (MK4) were shown to prevent lipid peroxidation in a dose-dependent way, with similar activity for both vitamers, and with warfarin counteracting the vitamin K effect [131]. Based on these results, dietary intake of vitamin K may together with other phytonutrients, strengthen cellular defence against oxidative stress.

## 4. Conclusions

It is currently known that the range of vitamin K action is far beyond the scope of blood coagulation. Although several available studies support a protective role for vitamin K in aging and age-related conditions such as cardiovascular disease, osteoarthritis and osteoporosis, the real evidence of the benefit of vitamin K supplementation is still controversial, since most of the available studies are observational and only a few randomized trials are currently available. Nevertheless, an important conclusion has been drawn relatively to its safe use as a health benefit, since no adverse effect or documented toxicity for K1 or MK4 and MK7 has ever been reported for individuals consuming higher amounts of vitamin K. Even when doses above the recommended daily allowance (RDA) of 75 micrograms vitamin K (Commission Directive 2008/100/EC) were used, no hypercoagulable state was observed. Based on this, a Tolerable Upper Intake Level is not available. 

Regardless of the available evidences clearly supporting a beneficial health effect of vitamin K, its underlying mechanism of action specialty regarding its novel and less explored functions as molecule with anti-inflammatory and antioxidant properties, is not yet known. This is particularly relevant in age-related diseases and in aging conditions such as functional decline, disability, sarcopenia and frailty, affecting the elderly in an aging society. In fact, aging societies represent a major economic challenge for health care systems, and diet supplements promoting healthy aging and improving the prognosis of age-related diseases are urgently needed to be implemented in clinical practice.

## Abbreviations

AVC	Aortic valve calcification
BMD	Bone mineral density
CAC	Coronary artery calcification
CaP	Calcium phosphate mineral
CHD	Coronary heart disease
CKD	Chronic kidney disease
CPPs	Circulating calciprotein particles
CRP	C-reactive protein
CVD	Cardiovascular disease
cGRP	γ-Carboxylated Gla-rich Protein
dp-ucMGP	Desphospho-uncarboxylated Matrix Gla Protein
EVs	Extracellular vesicles
GGCX	Gamma-glutamyl carboxylase
Gla	Gamma-carboxyglutamic acid residue
Glu	Glutamic acid residue
GRP	Gla-rich Protein
HD	Haemodialysis
IL-6	Interleukin 6
MGP	Matrix Gla Protein
MKs	Menaquinones
NF-κB	Nuclear factor kappa B
OA	Osteoarthritis
OC	Osteocalcin
RA	Rheumatoid arthritis
ROS	Reactive oxygen species
SASP	Senescence-associated secretory phenotype
TNF-α	Tumor necrosis factor alpha
ucGRP	Uncarboxylated Gla-rich Protein
ucMGP	Uncarboxylated Matrix Gla Protein
ucOC	Uncarboxylated Osteocalcin
VC	Vascular calcification
VKA	Vitamin K antagonist
VKDPs	Vitamin K-dependent proteins
VSMCs	Vascular Smooth Muscle Cells
